# Cognitive load in cyclists while navigating in traffic: Effects of static and dynamic route events on neural activity of cyclists measured by fNIRS

**DOI:** 10.1371/journal.pone.0339027

**Published:** 2025-12-19

**Authors:** Mehmet Baran Ulak, Fenja Heine, Aykut Eken, Pookhao Chinpongsuwan, Mario Boot, Karst T. Geurs, Funda Yildirim

**Affiliations:** 1 Department of Civil Engineering and Management, University of Twente, Enschede, the Netherlands; 2 brainboost, Munich, Germany; 3 Department of Psychology, Carl von Ossietzky University of Oldenburg, Oldenburg, Germany; 4 Department of Biomedical Engineering, TOBB University of Economics and Technology, Ankara, Türkiye; 5 Eindhoven University of Technology, Eindhoven, the Netherlands; 6 Faculty of Behavioural, Management and Social Sciences, University of Twente, Enschede, the Netherlands; National University of Sciences and Technology, PAKISTAN

## Abstract

Increased cognitive load during cycling can lead to safety issues, particularly during complex situations such as participating in motorised traffic or manoeuvring at intersections. Understanding the patterns of cognitive activity in cyclists across various events can provide valuable insights into cyclists’ cognitive load. Therefore, this study aims to investigate when cyclists undergo cognitive load changes during cycling and how these changes manifest in neural activity and connectivity. For this purpose, we analysed neural activity data collected during a real-life field experiment by a non-invasive portable method, namely Functional Near-Infrared Spectroscopy (fNIRS), sensitive to neural activity in the prefrontal cortex region. Findings indicate differences in cognitive load, as shown by varying oxygenation levels, during both static (i.e., presence of traffic lights, intersections, roundabouts, or crosswalks) and dynamic (i.e., presence of motorised vehicles, cyclists, pedestrians, or avoiding objects) route events. Neural activity patterns highlight how different road events elicit varying cognitive and neural demands in cyclists. Events like intersections and pedestrian encounters show dense connectivity, particularly in regions related to decision-making, attention, and motor planning, implying a high cognitive load on cyclists. Roundabouts and traffic light scenarios demonstrate intermediate connectivity, indicating the need for adaptive attention and action selection. This study contributes to understanding the underlying cognitive mechanisms during cycling in real-life conditions and the neural markers that can identify different route events encountered while cycling.

## 1. Introduction

Cost-effectiveness, environmental benefits, and health impacts of cycling motivate several countries to expand their cycling networks [1]. Nevertheless, the safety of cyclists is still a major concern for cycling to be an essential part of a well-functioning transport system. While cycling is generally safe, it occurs in a dangerous environment where motorists are a primary cause of severe crashes, along with single-bike crashes [1,2]. In addition to external factors such as participating in traffic, it was shown that increased cognitive load can lead to dangerous situations and potentially road crashes [3].

Cognitive load denotes the mental resources necessary to perform a specific task, representing the interaction of the human capacities and task demands [4]. Increased cognitive load hinders perception and attention during traffic participation [5]. The inherent complexity of urban roads places substantial demands on the attentional system, making cognitive load especially critical [3,6]. Route events that elevate cognitive load are often associated with pedestrians, parked cars, and higher traffic densities [7].

Despite the presence of studies focusing on motor-vehicle drivers, there is scarce research exploring the cognitive load in cyclists. Understanding the patterns of cognitive activity in cyclists across various events can provide valuable insights into cyclists’ cognitive load in complex situations. Although there are a few studies utilising fNIRS to investigate cognitive load in car drivers [4,6,7], there is no research on understanding the cognitive load in the context of cycling using fNIRS. Therefore, this study aims to investigate when cyclists undergo cognitive load changes during cycling and how these changes manifest in neural activity and connectivity. For this purpose, we analysed neural activity data collected during a real-life field experiment by a non-invasive portable method, namely Functional Near-Infrared Spectroscopy (fNIRS), sensitive to neural activity in the prefrontal cortex region. fNIRS is more resilient than alternatives such as the electroencephalogram (EEG) against external signals, making fNIRS a suitable tool for real-life field experiments.

We anticipated cyclists to experience increased cognitive load represented by neural activity (measured by elevated oxyhaemoglobin – HbO levels) during both static (i.e., presence of traffic lights, intersections, roundabouts, or crosswalks) and dynamic (i.e., presence of motorised vehicles, cyclists, pedestrians, or avoiding objects) route events. This increase is expected to be more pronounced in dynamic events since higher cognitive load has been found to coincide with dynamic route events involving pedestrians, parked cars, and other traffic participants such as moving vehicles and cyclists [6–8]. Furthermore, we expected to discover strong connections between various regions within the prefrontal cortex (PFC) while cycling, as previous studies have shown that connectivity within the PFC increases with cognitive load [9,10].

Consequently, this study investigates the fluctuations in neural activity patterns, as indicated by changes in oxygenated and deoxygenated haemoglobin levels, during cycling across various static and dynamic route events. We also examined the synchronised activation between different regions of the PFC through connectivity analysis. As such, this paper contributes to identifying the regions of the prefrontal cortex that present the highest cognitive activity and connectivity between these regions during static and dynamic route events. Our study helps advance our understanding of the underlying cognitive mechanisms during cycling in real-life conditions and on the neural markers that can identify different route events encountered while cycling, which can guide future human bike interaction applications and real-time neurofeedback applications to improve the safety of cyclists.

## 2. Cognitive load, neurological indicators, and measurement methods

### 2.1. Cognitive load and neurological indicators

Cognitive load in adults appears to involve multiple brain regions (prefrontal cortex, cingulate gyrus, temporal and parietal regions, and insula areas on both sides) [3,11]. Multiple brain regions are activated together as a network when cognitive load increases, which is called functional connectivity [12]. Examining functional connectivity during cognitive processes like cognitive load provides insights into how the brain’s networks reorganise to handle increased demands [13]. Research shows that the brain adapts its network organisation with increasing cognitive load, particularly involving the frontoparietal executive control network (FPN) [14]. Analysing these connectivity patterns not only reveals engagement during tasks but also predicts performance under cognitive load, as a more responsive network is linked to better outcomes [10,15,16].

An important brain area that is involved in the FPN network is the dorsolateral prefrontal cortex (DLPFC), which is part of the PFC [17]. Outside of this network, it was also found that connectivity between areas within the PFC, as well as between the PFC and the parietal cortex, increased with cognitive load [9,10]. When applied to driving, connections between the PFC, motor-related areas, the parietal cortex, vision-related areas, the thalamus, as well as the cerebellum can be found, indicating the involvement of various brain regions in cognitive load and driving [12].

### 2.2. Approaches to measure cognitive load

Different neuroimaging methods, such as electroencephalography (EEG) and functional magnetic resonance imaging (fMRI), can detect changes in cognitive workload [4]. However, in ecological contexts, functional near-infrared spectroscopy (fNIRS) is more suitable for measuring cognitive load compared to EEG and fMRI [4]. fNIRS measures changes in brain activation through oxygenated and deoxygenated haemoglobin concentrations in the blood [3]. Its portability and low sensitivity to motion artefacts make fNIRS ideal for investigating cognitive load in naturalistic settings without participant constraints or data quality compromises [6,17]. Studies exploring the relationship between driving and cognitive load using fNIRS observed changes in oxygenated and deoxygenated haemoglobin levels in the prefrontal cortex with increasing cognitive load [4,7]. Specifically, research employing fNIRS to investigate cognitive load found that heightened cognitive demands led to oxygenation changes in the left dorsolateral prefrontal cortex and increased brain activation in both the right and left prefrontal cortex [4].

### 2.3. Cognitive load and traffic tasks

Previous studies primarily examined cognitive load in the context of car driving in a simulation environment, revealing increased cognitive load during activities such as overtaking manoeuvres [3]. Furthermore, cognitive load rises during driving on demanding urban routes with numerous junctions and pedestrians, as compared to driving on straight roads with no pedestrians [6]. Increased cognitive load has also been linked to reduced event detection performance, as it diverts attention away from the forward scene and narrows the field of view, thereby impairing hazard detection [18,19]. Drivers under cognitive load found it challenging to recall interactions with roadway elements, including other traffic participants and road-related fixtures, following a driving session [19,20]. For example, they would be unable to remember passing by cyclists on the road or overlook certain traffic signs. Previous research also demonstrated that car drivers experiencing high cognitive demand consistently exhibited delayed braking responses, which is a critical factor in road safety [18,20]. Interestingly, increased cognitive load has been found to enhance driving performance in lane-keeping situations [5]. This highlights that the relation between cognitive load and driving performance is not straightforward [5]. We expect that car driving and cycling are fundamentally different in terms of cognitive activity and loading; however, to the authors’ knowledge, there is no research focusing on the cognitive loading in cyclists through fNIRS.

## 3. Experimental setup and data

In this study, we utilised a previously collected fNIRS dataset [21] in conjunction with video footage captured by an action camera (GoPro HERO 9) [22]. Hemodynamic responses of participants were collected using fNIRS during a cycling task along a pre-defined route. The study involved 17 (Gender female/male: 6/11, age = 23 ± 4.1, range: 19–35) participants who were selected based on the absence of psychoactive drug use, absence of brain-related disorders, and competence in cycling, such as being able to remove one hand from the steering while cycling. Informed consent was obtained from all participants.

### 3.1. Experimental setup

Participants cycled a 7.5 km route through diverse environments (please see [22] for details of the route). The cycling phase of the experiments took approximately 30 minutes on average; however, the exact trip duration slightly varies depending on the speed of the individual riding the bike. A portion of the route exposed participants to motorised traffic, with cycling lanes running parallel to the road, sometimes without physical barriers. This section included frequent traffic stops, intersections, and interactions with other cyclists. Another segment of the route involved dedicated bicycle pathways away from the main road, leading through natural surroundings. Simultaneously, an action camera (GoPro HERO 9) was used to record the cycling route from a forward-facing perspective. Participants were instructed to abstain from alcohol and psychoactive drugs at least 24 hours before their trial, for safety reasons and to avoid potential confounding factors such as unnatural neural responses. Participants were also instructed to bring their own pedal-powered bicycle; electric bicycles were not permitted for use during the trial.

At the start of the trial, the fNIRS device was placed on the participant’s head and connected via Bluetooth to a laptop running OxySoft [23]. The received near-infrared light power was checked before the experiment to ensure that all channels received adequate light. Subsequently, the fNIRS device was disconnected from OxySoft in preparation for offline measurement. Participants were then escorted outside to their bicycles, where the action camera (GoPro HERO 9) and a mobile phone (Apple iPhone XR) were mounted on their bicycles. The mobile phone was equipped with the PIEL Survey experience sampling app (pielsurvey.org), which had been preprogrammed for the experiment. After the initialisation of camera recording and fNIRS measurement, participants were instructed to remain still for approximately two minutes to establish a baseline measurement before starting to cycle. All data collection devices, camera, fNIRS and mobile phone were synchronised based on the internal time unit of devices, which were checked and adjusted before experiments.

### 3.2. fNIRS channel configuration

In this study, the data were collected from the entire prefrontal cortex (please see [24] for regions of PFC). For in-depth analysis of cognitive loads, we focused on the Dorsolateral prefrontal cortex (DLPFC) in both hemispheres. The DLPFC was chosen as the region of interest due to its frequent association with cognitive load. Previous studies have consistently linked cognitive load with ΔHbO (the change in the oxyhaemoglobin level) levels in the DLPFC [25–27] with significantly higher blood oxygenation levels observed during tasks involving increased cognitive load [28].

Each hemisphere of the DLPFC appears to be more dominant for different types of cognitive workload; for instance, the left DLPFC is more implicated in tasks requiring memory load, while the right DLPFC is dominant during vigilance-related tasks [4]. Optodes were positioned over both sides of the DLPFC, as cognitive workload affects both hemispheres [29]. Each hemisphere was represented by two channels corresponding to the DLPFC. For the right DLPFC, channels between optodes Tx2, Rx1 and Rx3 (channels 3 and 4) were analysed (please see S1 Appendix for channel configuration). Likewise, for the left DLPFC, channels between optodes Tx10, Rx7 and Rx8 were examined (channels 26 and 27). Please refer to Figure A2 and Table A in the S1 Appendix for channels and optode placement.

### 3.3. Data acquisition

fNIRS signals were collected using the Artinis Brite MKII [30], continuous wave fNIRS system. 760 and 850 nm near-infrared light was sent to the scalp by using emitters, and absorbed and scattered light was detected by using detectors and sampled at a 50 Hz sampling rate. The setup included 18 optodes (i.e., sensors measuring oxygenation levels), comprising 10 emitters and 8 detectors, resulting in 27 measurement channels. Optodes were placed over the forehead to capture the change in levels of oxyhaemoglobin (ΔHbO) and deoxyhaemoglobin (ΔHb) signals in the prefrontal cortex related to cognitive activity during the experiment. Raw light intensity signals were transmitted via Bluetooth to the recording computer and recorded by using OxySoft software (version 3.5.15.4. [30]).

### 3.4. Data preprocessing

#### 3.4.1. Static and dynamic route events.

The video recordings captured with the action camera were reviewed, and events observed during the participants’ trials were documented. The video data were accessed between 10 November 2023 and 23 June 2024. From this list of events, those that occurred frequently across multiple participants were grouped based on similarity, where applicable (e.g., the Motorised Vehicle event encompassed both cars and motorcycles), and selected as the final event codes. Additionally, the event codes were categorised into static and dynamic road events (Table 1). Static events encompassed road infrastructure elements, while dynamic events included moving elements. The video recordings were transcribed according to these event codes. This process was carried out for all events captured during the video recording of the route, for all participants. Intervals during which participants cycled without encountering any type of intersections or moving objects were labelled as Baseline.

**Table 1:** Classification by static or dynamic route events.

Static route event	Dynamic route event
Traffic Light (TL)	Motorised Vehicles (MV)
Intersection (I)	Cyclist (C)
Roundabout (R)	Pedestrian (P)
Crosswalk (CW)	Avoiding Object* (AO)

*Avoiding Object indicates instances where participants encountered an object blocking their path, requiring them to move around it.

#### 3.4.2. fNIRS data processing.

The fNIRS data recorded during cycling sessions were converted to.snirf files (Shared Near InfraRed File Format) using OxySoft, and data analysis was performed using Homer3 [31], a MATLAB application designed to analyse fNIRS data. In Homer3, all channels were visually inspected for every participant and channels with poor signal quality, by considering heart rate activity, were removed manually. Two participants were excluded from further analysis due to poor data quality or missing data. The data were accessed between 10 November 2023 and 23 June 2024.

After visually inspecting and pruning the channels, raw light intensity data were converted to optical density (OD). To remove motion artefacts, a wavelet-based motion correction was applied to the data [32]. A low-pass filter with a cutoff frequency of 0.5 Hz was used to reduce high-frequency instrument noise and physiological noise, such as fast cardiac oscillations [3]. Subsequently, OD data were then converted to oxyhaemoglobin (ΔHbO) and deoxyhaemoglobin (ΔHb) concentration data by using the Modified Beer-Lambert Law [33].

The concentration data were segmented into epochs, each corresponding to the time stamps of the eight route events and the Baseline, which were determined based on the video recordings. For the HbO analysis, after epoching, block averaging was performed over a five-second interval, spanning one second before event onset to four seconds after event onset. We chose 5 seconds because it captures the trend of the BOLD signal, which peaks between 3 and 7 seconds after stimulus onset, ensuring a comprehensive view of the hemodynamic response despite varying event durations [34–36].

In our analyses, we focused on oxygenated haemoglobin (ΔHbO) values, due to having a higher Signal-to-Noise ratio than ΔHb, and they have demonstrated a stronger correlation with blood flow compared to ΔHb, thus serving as a more reliable marker of hemodynamic activation [17]. We extracted the ΔHbO hemodynamic response function values from channels positioned over the DLPFC to understand the similarity between the events.

## 4. Methodology

The methodology of this study is composed of four parts: 1) analysis of oxygenation levels, 2) dynamic time warping analysis, 3) analysis of graph metrics, and 4) connectivity analysis. The experimental setup and study were reviewed and approved by the Natural Sciences & Engineering Sciences (NES) Ethics Committee of the Faculty of Engineering Technology of the University of Twente (Request number: 230212).

### 4.1. Analysis of the effects of events on the oxygenation levels

To understand the changes in brain activity patterns during cycling across various events and the relationship between route events and changes in cognitive load, we analysed the oxygenation levels in the PFC regions of participants. For this purpose, a five-second time frame was extracted, which covers one second before and four seconds after an event started (based on video recording assessment). These signals were composed of 250 measurements (50 measurements per second), which were plotted to illustrate the differences in the temporal variation of the oxygenation levels. Furthermore, we conducted t-tests to compare the average oxygenation levels for each event for each moment of measurement (250 in total) to identify the moments that any two events (e.g., crosswalk and presence of motor vehicle) produced statistically significantly different oxygenation levels.

### 4.2. Dynamic time warping

The Dynamic Time Warping (DTW) method is used to understand the similarity between time series signals, that is, between oxygenation levels produced when encountering cycling route events. We employed DTW to understand whether it is possible to distinguish route events based on the signals they caused in each optode, measuring the oxygenation level in a specific brain region. Moreover, DTW is used to understand whether there are similarities among signals of different participants for the same kind of route event.

DTW is a similarity measurement algorithm for two time series [37]. DTW is used to perform an optimum sequence alignment between two time series and detect identical shapes between temporally varying time series by performing the elastic transformation. For instance, let $S_{\text{1}}=[s_{\text{1},\text{0}},s_{\text{1},\text{1}},s_{\text{1},\text{2}}\ldots \ldots .s_{\text{1},N-\text{1}}]$ and $S_{\text{2}}=[s_{\text{2},\text{0}},s_{\text{2},\text{1}},s_{\text{2},\text{2}}\ldots \ldots .s_{\text{2},\;\;N-\text{1}}]$ be two time series that have the same length N and M is a distance matrix with N x N dimensions. For every index of M, Euclidean distance is estimated between $s_{\text{1},i}$ and $s_{\text{2},\;j}$ as stated equation 1;


$$M_{ij=}d(s_{\text{1},\;i},s_{\text{2},j})$$ (1)

Having estimated $M_{ij}$ that represents the distance for each sample pair, the DTW algorithm attempts to find the shortest path between $S_{\text{1}}$ and $S_{\text{2}}$ by searching from the index (0,0) to (N−1,N−1). The shortest path on this M matrix is called the Warping path and is represented as W:


$$W=[w_{\text{0}},w_{\text{1}},\ldots \ldots ..w_{k}]$$ (2)

W is generated by utilising linear programming. First, Euclidean distance $d(s_{\text{1},\;i},s_{\text{2},j})$ is found and defined as a cost value to estimate the minimum distance between two time series. After estimating the initial Euclidean distance, the minimum value of the neighbouring cells (M(i−1,j−1), M(i−1,j), M(i,j−1))) is selected and M(i,j) is calculated by summing of $d(s_{\text{1},\;i},s_{\text{2},j})$ and the minimum value as shown in Equation 3.


$$M(i,j)=\;d(s_{\text{1},\;i},s_{\text{2},j})+min(M(i-\text{1},j-\text{1}),\;M(i-\text{1},j),\;M(i,j-\text{1}))$$ (3)

Let the indices of the warping matrix be p and r for one time series, and x and y for the other one, *t*^th^ and *t-1*^th^ sample represents as $w_{t}=(p,r)$ and $w_{t-\text{1}}=(p^{\prime },r^{\prime })$ in our warping path, and this warping path must satisfy four main constraints:

- Monotonicity constraint: Indices in the warping path should either stay the same or increase in the time domain. This prevents repeating the warping path. Indices ensure the following condition, as shown in equation 4:

$$p\geq p^{\prime }\;\&\;r\geq r^{\prime }$$ (4)

- Continuity constraint: The warping path advances one step at a time. The index difference between $p\;-\;p^{\prime }$ and $r\;-\;r^{\prime }$ should be less than or equal to one as shown in equation 5.

$$p-p^{\prime }\leq \text{1}\;\&\;r-r^{\prime }\leq \text{1}$$ (5)

- Boundary constraint: The warping path starts from value M(0,0) and ends in value M(N−1,N−1).
- Warping window constraint: The alignment path onto the distance matrix M is not expected to drift from the diagonal. For warping window length l

|p−r|>l & |x−y|>l (6)

Finally, to determine whether different participants have similar oxygenation levels for a given event and to illustrate whether different events caused substantially different oxygenation levels, we utilised the sum of squared differences from the mean (SSDM) for the calculated DTW values. SSDM calculated the difference observed for a given set of measurements compared to the mean observation. Therefore, larger SSDM values indicate that there is a large variation among measurements, while smaller SSDM values imply that measurements are close to the mean measurements.

We expected that the SSDM values calculated for oxygenation levels among participants for a given event would be substantially smaller than the SSDM values calculated for the oxygenation levels measured among events. Such a finding would imply that participants have similar neurological reactions to different events, while different events produce distinct neurological patterns that can be distinguished regardless of the participant’s characteristics. SSDM formulation is shown in equation 7:


$$SSDM_{i}=\sqrt{\sum_{j=\text{1}}^{N}{\left({DTW}_{ij}-\overset{―}{DTW}\right)}^{\text{2}}}$$ (7)

where $SSDM_{i}$ is the sum of squared differences from the mean of the measurements under consideration (e.g., DTW value of oxygenation for crosswalk event for all participants), ${DTW}_{ij}$ is the calculated DTW value for the oxygenation level per two entities i and j (e.g., two participants or two events), the $\overset{―}{DTW}$ is the average DTW of oxygenation level for all entity pairs (for all participant pairs for a given event to identify inter-participant variation or all event pairs to identify inter-event variation), and N is the number of entities (e.g., number of participants or events). We calculated SSDM values for each channel to identify the most feasible channels that can be used to distinguish different events based on oxygenation levels and see the inter-participant variation. Statistical analyses were conducted using the R programming language within RStudio.

### 4.3. Graph metrics

Graph theory and connectivity have emerged as key concepts in neuroscience for uncovering the brain’s network properties [38–40] and have been successfully applied to fNIRS data [41]. To analyse the identified connectivity of different regions of the PFC, we employed four different graph metrics: modularity, global clustering, global efficiency, and small-worldness [42]. The first two metrics address the connectivity or clustering tendency of the nodes in the network, while the latter two measure the ease of information exchange among nodes. Modularity is a metric measuring the density of the connections between the nodes within and outside the separate modules in the network. That is, high modularity indicates a high connectivity between nodes within modules but low connectivity among nodes belonging to separate modules. The global clustering (or transitivity) measures the degree of clustering between nodes of the network. High clustering indicates that nodes tend to cluster and create communities where nodes of these communities are densely connected internally.

The global efficiency of the network measures the closeness of the nodes in the network to each other. The closer the nodes are to each other, the easier to reach from one node to the other. Therefore, the metric measures the effectiveness of the overall network in terms of communication or information flow from one node to another. Small-worldness is also a feature of networks that defines the ease of reaching one node from another. Networks that have nodes that have fewer neighbours but still can be accessed through a few edges are considered small networks.

We used these metrics to define the cognitive networks that appear during static and dynamic events. The metrics hint at the cognitive loading and communication density between the parts of the prefrontal cortex. Therefore, they can reveal the cognitive functioning during these events. Please refer to Rubinov and Sporns [42] for further details and mathematical formulations of these metrics. We used the “qgraph” and the “igraph” packages on the R-Studio platform to calculate the values of these metrics [43].

### 4.4. Connectivity analysis

For the connectivity analysis, we used the block-averaged concentration data, which are the averages of the pre-processed data. To visualise the connectivity between the different channels, the Montreal Neurological Institute (MNI) coordinates of the channels were extracted from OxySoft by digitising the optodes and creating a topography. The links and thickness of the links in the identified graphs illustrate the varying strengths of connectivity between channels and show the location of each channel within the PFC. To analyse the connectivity between the channels (i.e., regions in the prefrontal cortex), we used a graph learning approach, namely graphical lasso estimation (GLE).

A graphical model is a graph representation of the conditional dependence structure of the features in the dataset. In other words, GLE can reveal the underlying covariance structure among random variables, and this structure can be used to discover latent relationships between features associated with a phenomenon [44]. GLE was previously used in several studies to predict gene regulatory networks [45] and to explore brain connectivity [46].

GLE was also used in the transport research domain to conduct a graphical analysis of psychological factors affecting driver behaviour [47] and in traffic safety to analyse the relationships between factors contributing to crashes [48]. For example, Ulak and Ozguven [48] employed the GLE in a similar manner but used it to understand the relationships between factors associated with traffic crashes.

The graphical lasso estimation (GLE) estimates a sparse graph based on the inverse of the covariance matrix (also called a precision matrix) of a data matrix with n observations and p features, with mean μ and covariance Σ. Considering that the empirical covariance matrix of this data matrix is S, the graphical lasso estimator can be obtained after the following steps [48,49]:


$$S=\frac{\text{1}}{n}\sum_{i=\text{1}}^{n}\left(x_{i}-\mu \right){\left(x_{i}-\mu \right)}^{T}$$ (8)

where $x_{i}$ is the $i^{th}$ observation in the data matrix $X=(x_{\text{1}},\;x_{\text{2}},\ldots ,\;x_{n})$ and μ is the mean. The likelihood function of the multivariate normally (MVN) distributed data matrix can be derived as follows:


$$L=\prod_{n=\text{1}}^{n}N(x_{i},\;\Sigma )=\prod_{n=\text{1}}^{n}\left\{\frac{\text{1}}{(\text{2}\pi {)}^{\frac{p}{\text{2}}}{det\;det\;(\Sigma )\;}^{\frac{\text{1}}{\text{2}}}}e^{\frac{\text{1}}{\text{2}}{\left(x_{i}-\mu \right)}^{T}\Sigma^{-\text{1}}\left(x_{i}-\mu \right)}\right\}$$ (9)

where N(.,.) is the multivariate normal distribution of X, Σ is the covariance matrix, and $\Sigma^{-\text{1}}$ is the inverse covariance matrix. The loglikelihood function of this likelihood function can be written as follows:


$$\;log\;(L)\;\;=-\frac{n}{\text{2}}\left(p\;log(\text{2}\pi )\;+log\;\left(det\;(\Sigma )\;\right)\;\right)-\frac{\text{1}}{\text{2}}\sum_{i=\text{1}}^{n}{\left(x_{i}-\mu \right)}^{T}\Sigma^{-\text{1}}\left(x_{i}-\mu \right)$$ (10)

Equation 10 can be further simplified by removing constant terms:


$$log\;(L)\;\propto -log\;\left(det\;(\Sigma )\;\right)\;-\frac{\text{1}}{n}\sum_{i=\text{1}}^{n}{\left(x_{i}-\mu \right)}^{T}\Sigma^{-\text{1}}\left(x_{i}-\mu \right)$$ (11)

And, finally, Equation 11 can be rewritten as follows:


$$log\;(L)\;\propto log\;\left(det\;\left(\Sigma^{-\text{1}}\right)\;\right)\;-tr\left(\Sigma^{-\text{1}}\cdot \frac{\text{1}}{n}\sum_{i=\text{1}}^{n}{\left(x_{i}-\mu \right)}^{T}\left(x_{i}-\mu \right)\right)$$ (12)

where tr(.) is the trace (the trace of an n-by-n square matrix is the sum of the main diagonal elements of that matrix) and $\Sigma^{-\text{1}}$ is the inverse covariance matrix. Combining with the equation 8, equation 12 becomes:


$$log\;(L)\propto \;log\left(det\left(\Sigma^{-\text{1}}\right)\;\right)\;-tr\left(S\cdot \Sigma^{-\text{1}}\right)$$ (13)

The GLE method utilizes $l_{\text{1}}$ norm penalty to maintain sparsity [50]. The $l_{\text{1}}$ norm is the summation of the absolute values of the matrix elements. Considering the $l_{\text{1}}$ penalty, the graphical lasso estimator based on equation 13 becomes:


$$\hat{\Sigma^{-\text{1}}}=argmin\left(tr\left(S\cdot \Sigma^{-\text{1}}\right)-log\;log\;\left(det\;det\;\left(\Sigma^{-\text{1}}\right)\;\right)\;+\lambda {\left|\left|\Sigma^{-\text{1}}\right|\right|}_{\text{1}}\right)$$ (14)

where λ is the tuning parameter, ${\left||\Sigma^{-\text{1}}|\right|}_{\text{1}}$ is $l_{\text{1}}$ norm of $\Sigma^{-\text{1}}$, and S is the empirical covariance matrix (equation 14). Equation 14 shows a convex optimisation problem for which convex optimisation algorithms can be used to solve [49,51]. Studies by [49,52,53] provide a comprehensive overview of the theory and application of graphical lasso estimation [49,52,53]. We estimate the graphical lasso using the “huge” package of R software [54]. For this purpose, we applied a nonparanormal transformation to the data [53]. For the parameter selection, we used the rotation information criterion (RIC) approach and found the optimal λ value [55,56], which resulted in the optimal graph.

## 5. Results

### 5.1. Time series analysis

**Fig 1:**
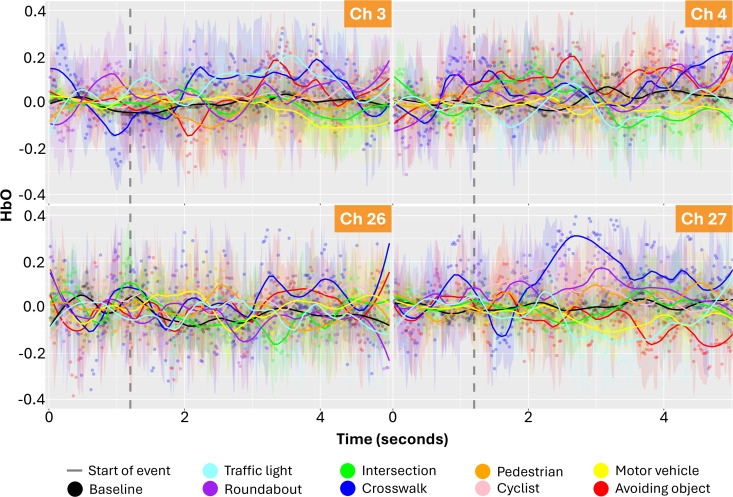
Time series plots of the oxygenation levels (micromolar - µM concentration change) at the brain regions (DLPFC channels) based on the static and dynamic events (dots are the average HbO level from all participants and lines are the smoothed curve showing the general trend): raw oxygenation levels, b) oxygenation levels with respect to Baseline.

**Fig 2:**
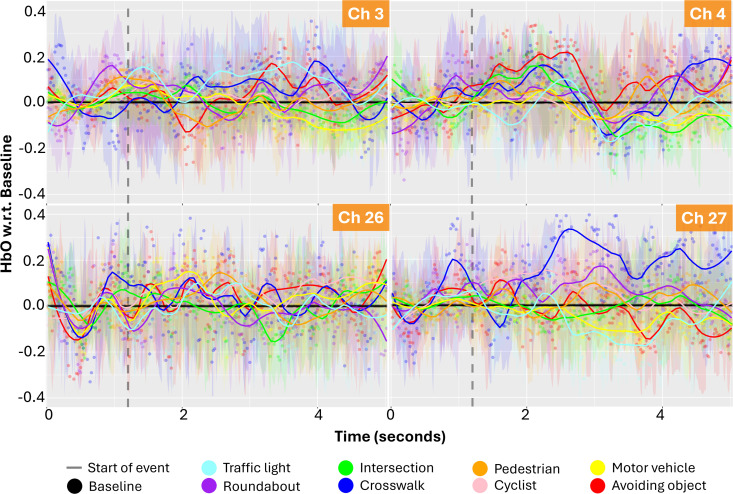
Time series plots of the oxygenation levels (micromolar - µM concentration change) at the brain regions (DLPFC channels) based on the static and dynamic events (dots are the average HbO level from all participants and lines are the smoothed curve showing the general trend): oxygenation levels with respect to Baseline.

Time series plots of the oxygenation levels at the brain regions (channels) based on the static and dynamic events show that fluctuations vary depending on the event. Figs 1 and 2 shows the HbO signals of different route events in four different channels (3, 4, 26, 27) corresponding to the dorsolateral prefrontal cortex (DLPFC) regions. In these figures, the values represented by dots show the average value for all participants for a given route event and a moment (1/50 seconds) in 5 seconds. The hemodynamic responses of participants starting from the moment of the beginning of each route event were extracted, and the average HbO and its standard deviation were calculated for each moment. The colour-shaded bandwidths are the 90% confidence interval of these mean estimates. The lines show the smoothed averages, which are provided to illustrate the trend of change using the locally estimated scatterplot smoothing function.

**Fig 3:**
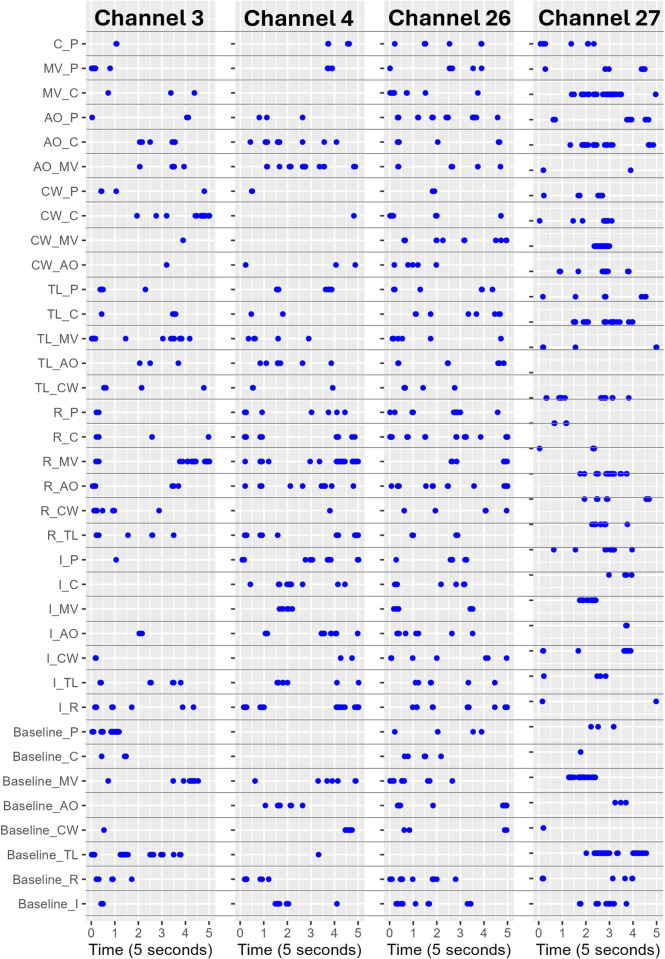
Statistically significant differences between HbO levels at channels in DLPFC for different events at each moment based on a t-test and a significance level of 0.05. Each blue dot represents a moment where the difference is statistically significant. The row names stand for the pair of events that are compared (e.g., I_C stands for the Intersection and Cyclist comparison).

In Figs 1 and 2, baseline measurements (black line) fluctuate substantially less than route events, indicating that route events are actually causing different neural activity patterns than the baseline activity. Furthermore, Fig 3 shows the t-test analysis results indicating whether the HbO signals (for channels 3, 4, 26, 27) at a given moment differ from event to event. Each blue dot in Fig 3 denotes a moment where two events (e.g., motor vehicle-MV vs. cyclist-C) produced statistically significantly different oxygenation levels. These pairwise between-event comparisons in time-series signals exhibit a strong distinction between different events, as indicated by the count of significant pairs (Fig 3). This means that the HbO signals in these channels can be used to distinguish the events encountered by the cyclists. Moreover, a lack of expected signal may indicate a distraction or a cyclist’s disregard for a certain event (e.g., presence of a motor vehicle).

### 5.2. Differences between channels in identifying the events

One of the objectives of this research was to identify the regions of the prefrontal cortex that present the highest variation in oxygenation levels between static and dynamic events (Table 1). Each optode of the fNIRS measures oxygenation levels, which depend on the events encountered by the participants. These measurements can be plotted as time series information, as shown in Fig 3. Since the preliminary analysis alone does not allow identifying optodes that produced distinguishable signals, we used the DTW approach to identify the difference between these signals. As a result, the DTW approach provided the degree of similarity or difference based on the oxygenation measurements of the channels. This analysis helped us to identify the channels that are the most feasible in distinguishing the events.

**Fig 4:**
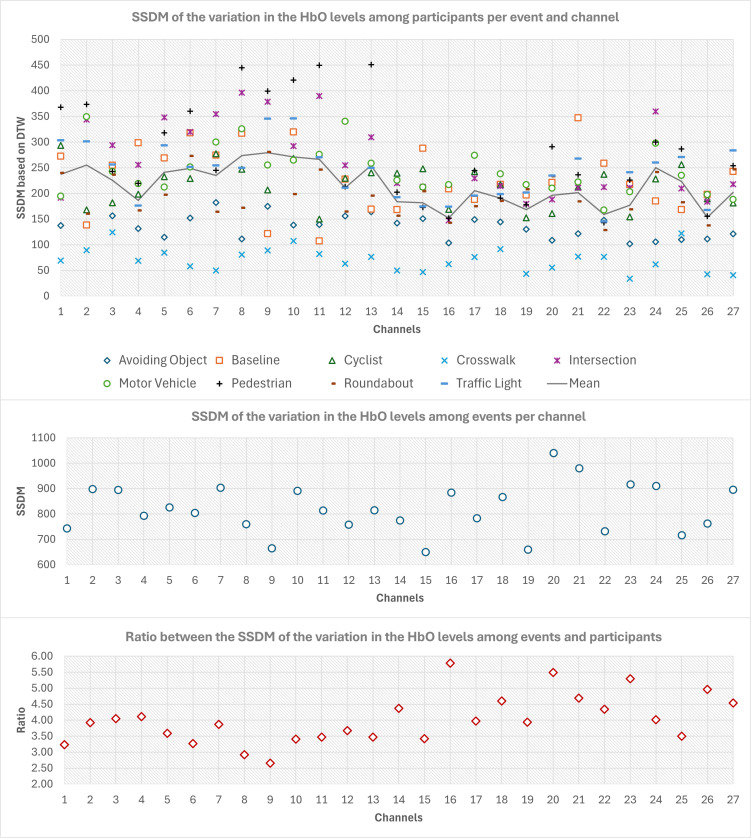
Top row) SSDM based on DTW for each participant. Middle row) SSDM based on DTW for each channel. Bottom row) Ratio of SSDMs based on DTW, calculated as the variation among channels divided by the variation across participants.

Variations in oxygenation levels across participants (Fig 4 top row), reflecting the signal reliability for cognitive load measurement, showed SSDM values ranging from 153 to 262. Channels with closely aligned SSDM values (i.e., within a narrow range) suggest greater similarity in their response profiles, implying consistency in signals across participants. Looking into Fig 4 (middle row), Channels 1, 5, 6, 8, 9, 14, 18, and 26 exhibit SSDM values ranging from approximately 650–775, indicating a relatively consistent response pattern across events. This similarity suggests that these channels might be part of a homogenous network or reflect similar cognitive processes. These channels are less likely to serve as neuromarkers for different events, as their uniformity implies limited differential sensitivity to varying cognitive loads. In contrast, channels such as 2, 10, 20, and 21 display significantly higher SSDM values (ranging from approximately 894–1040), deviating from the values seen in more consistent channels. This variability implies that these channels may respond differently to certain cognitive events, potentially due to region-specific brain activity. As such, these channels could serve as neuromarkers, providing unique signal profiles that differentiate between different cognitive states or conditions across route events. Fig 4 (bottom row), which shows the ratio between the values in Fig 4 (middle and top rows) shows that the most promising channels to distinguish events can be channels 16, 18, 20, 21, 23, 26, and 27.

### 5.3. Resultant connectivity

**Fig 5:**
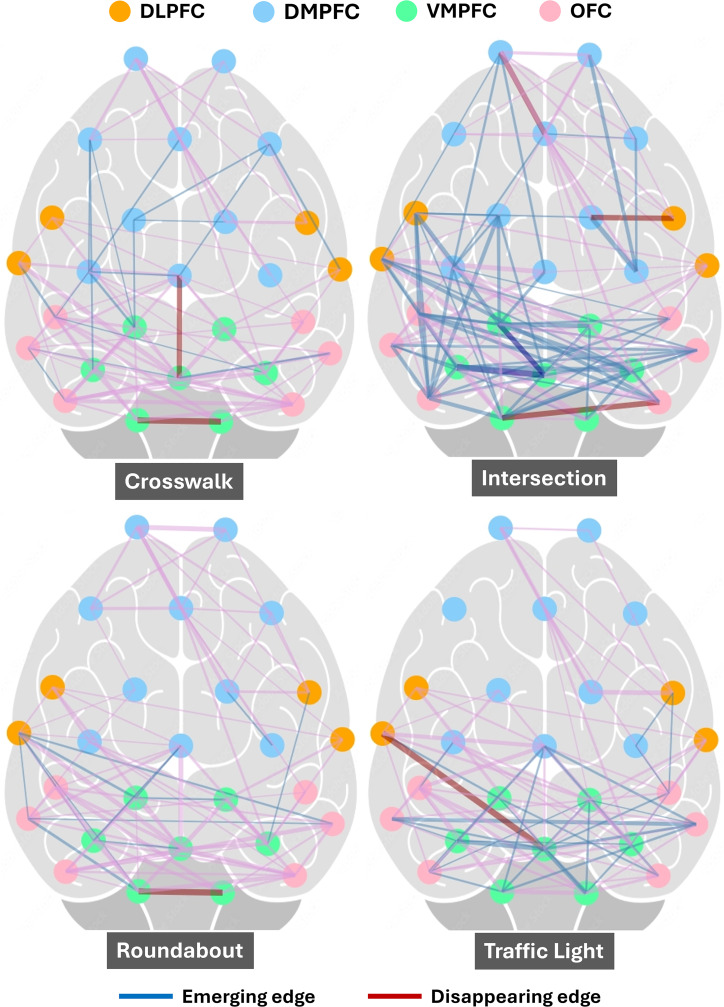
Connectivity within regions of the prefrontal cortex during different static events, compared to baseline. Thicker connecting lines indicate stronger connectivity between regions based on estimated covariance values. The emerging and disappearing edges highlight dynamic neural network adaptations depending on situational complexity.

**Fig 6:**
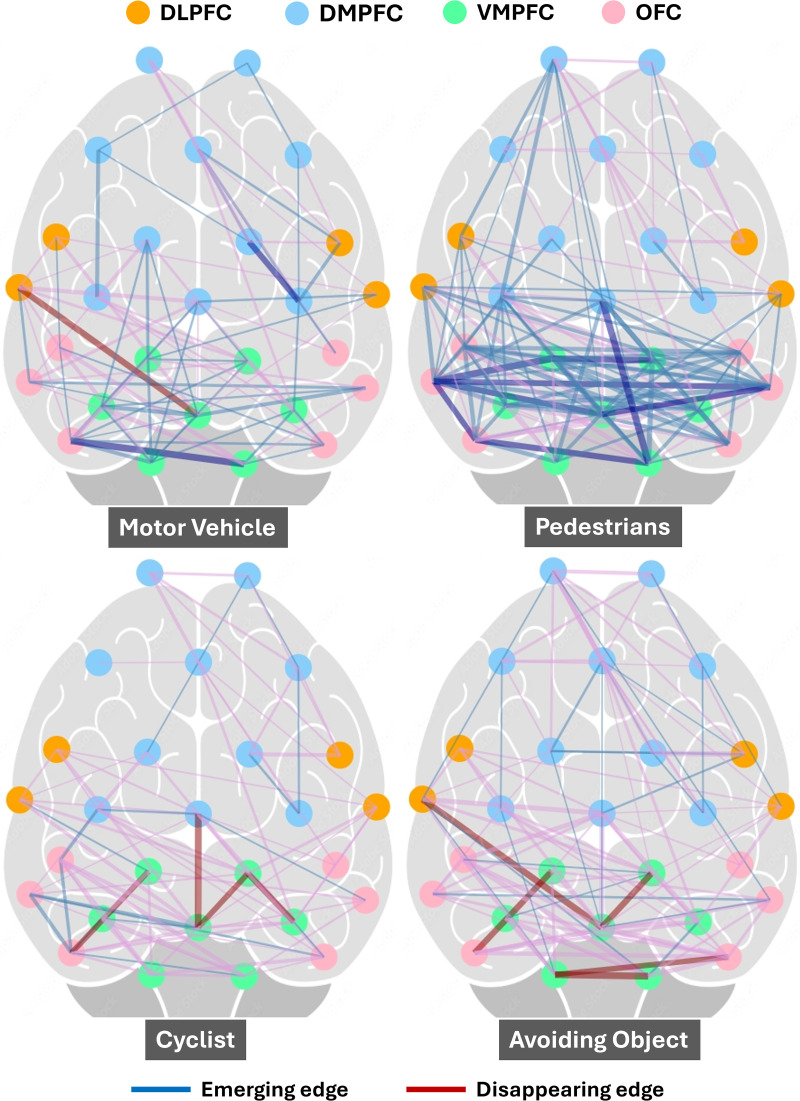
Connectivity within regions of the prefrontal cortex during different dynamic events, compared to baseline. Thicker connecting lines indicate stronger connectivity between regions based on estimated covariance values. The emerging and disappearing edges highlight dynamic neural network adaptations depending on situational complexity.

Using graphical lasso analysis, we examined the connectivity patterns during different traffic events compared to the baseline brain state, as measured by fNIRS (Figs 5 and 6). The findings revealed the strongest connections between specific brain regions. To observe the event-specific network dynamics, the connectivity matrices for the baseline condition were subtracted from each event for visualisation (Figs 5 and 6). Please refer to S1 Appendix (Figure A1) for the actual connectivity plots.

The brain connectivity patterns in Figs 5 and 6 reveal distinct neural activation across different cycling events. Intersection and Pedestrian events exhibit dense and widespread connectivity, particularly involving Dorsolateral Prefrontal Cortex (DLPFC) and Dorsomedial Prefrontal Cortex (DMPFC) regions. Similarly, Roundabout and Traffic Light events show notable connectivity, with Orbitofrontal Cortex (OFC) and Ventrolateral Prefrontal Cortex (VMPFC) connections. Motor Vehicle event displays moderate connectivity, emphasising reactive decision-making with specific prefrontal regions like OFC and DLPFC. Avoiding Object involves targeted, sparse connectivity, primarily between Ventromedial Prefrontal Cortex (VMPFC) and DMPFC. Crosswalk and Cyclist events demonstrate localised connectivity, particularly within OFC.

The VMPFC demonstrates stronger within-region connectivity during activities such as avoiding objects, navigating intersections, and responding to traffic lights. This indicates its involvement in integrating sensory inputs, assessing potential risks, and making value-based decisions in situations that require quick and precise judgment. The increased connectivity between the OFC and the VMPFC during encounters with moving vehicles, pedestrians, and roundabouts highlights the importance of these regions in high-stakes decision-making. This connectivity likely supports evaluating multiple options and outcomes while navigating.

Stronger within-region connectivity in the OFC while observing other cyclists suggests a heightened state of attentional focus and environmental monitoring. The OFC is essential for weighing immediate environmental cues and guiding action execution based on those inputs. The engagement of OFC-DLPFC and DMPFC-VMPFC connectivity at crosswalks underscores the integration of executive functions, such as sustained attention and goal-directed behaviour, with real-time decision-making. This is crucial for managing transitions between observing the environment and executing precise actions, such as yielding or crossing safely.

Events such as *intersections* and encounters with *pedestrians* exhibit the highest density of emerging edges. These patterns involve connections predominantly within the DLPFC and VMPFC, reflecting increased cognitive demands. The networks activated during these events support decision-making, strategic planning, attention shifting, and multitasking in dynamic and complex environments.

Moderate levels of connectivity are observed during events like *roundabouts*, *traffic lights*, and navigating alongside other *cyclists*. These events show an intermediate number of emerging edges, indicating activation of regions associated with task-switching, spatial navigation, and inhibitory control. *Roundabouts*, for instance, require adaptation to continuous movement, while traffic lights and cyclist interactions involve selective attention and action planning in structured yet dynamic contexts.

*Avoiding objects* and encounters with *motor vehicles* shows more focused connectivity patterns, primarily involving the DMPFC and motor-planning regions. These tasks involve limited emerging edges, suggesting reliance on pre-established reflexive or automated pathways for rapid decision-making and action execution. These scenarios are less cognitively demanding compared to interactions requiring more complex situational awareness. *Crosswalk* presents moderately distributed connectivity, reflecting activation of attentional and decision-making pathways in controlled pedestrian environments. Compared to the baseline condition, crosswalk events involve a slight increase in emerging edges, but connectivity remains less complex than in dynamic events like intersections. Baseline connectivity serves as a reference, showing minimal modulation or adaptive shifts.

### 5.4. Resultant network metrics

The graph theory analysis across conditions also revealed similarities and differences in modularity, global clustering, global efficiency, and small-worldness (Table 2). Modularity values, while generally consistent across conditions, were highest in Traffic Light (0.18) and Motor Vehicle (0.16), suggesting greater network specialisation compared to Crosswalk, which had the lowest modularity (0.05). This similarity in modularity among most conditions, with Crosswalk as an outlier, points to comparable network organisation within clusters across conditions.

**Table 2:** Graph metrics of identified graphs.

	Baseline	Traffic Lights	Roundabout	Intersection	Crosswalk	Pedestrian	Cyclist	Motor Vehicle	Avoiding Object
Modularity	0.10	0.18	0.13	0.13	0.05	0.10	0.15	0.16	0.12
Global clustering	0.49	0.65	0.46	0.51	0.50	0.66	0.46	0.52	0.40
Global efficiency	8.58	7.75	12.95	5.89	14.61	6.35	10.59	8.25	16.37
Small-Worldness	1.53	1.46	1.33	1.31	1.55	1.21	1.52	1.57	1.35

Global clustering showed a pattern of similarity between Motor Vehicle and Traffic Light (0.66 and 0.65), indicating densely connected networks in these conditions. By contrast, Avoiding Object (0.40) and Crosswalk (0.50) displayed lower clustering, signifying sparser interconnections within these networks. Global efficiency, with Avoiding Object at its highest (16.37), suggests greater information flow across this network, while lower values in Intersection (5.89) and Motor Vehicle (6.35) highlight dissimilarities due to restricted efficiency. Most conditions were moderately similar in small-worldness (1.31–1.57), with Motor Vehicle and Crosswalk reaching the highest values (1.57 and 1.55), pointing to robust small-world organisation, while Avoiding Object (1.35) and Intersection (1.31) presented the lowest, marking these conditions as less tightly organised in comparison.

*Modularity* reflects the extent to which the brain’s network is organised into distinct modules or communities. Higher modularity during Traffic Light (0.18) and Motorised Vehicles (0.16) suggests strong compartmentalisation, potentially indicating specialised processing. In contrast, lower modularity during Crosswalk (0.05) suggests a more integrated network, likely facilitating coordination between regions for dynamic decision-making and action execution.

*Global clustering* indicates the tendency of the brain to form local clusters or tightly connected groups. Pedestrian (0.66) and Traffic Light (0.65) show the highest clustering, which may signify localised processing for maintaining attention to stationary and predictable cues. Conversely, Avoiding Object (0.40) and Baseline (0.49) show lower clustering, reflecting reduced localised activity when no specific task is engaged.

*Global efficiency* measures the ability of the brain network to integrate information across distant regions. The highest efficiency occurs during Avoiding Object (16.37) and Crosswalk (14.61), highlighting the need for rapid, coordinated decision-making and action during these complex tasks. The lowest efficiency during Intersection (I) (5.89) and Pedestrian (6.35) suggests more localised, less integrated processing.

*Small-worldness* reflects an optimal balance between local specialisation and global integration. Motorised Vehicles (1.57) and Crosswalk (1.55) demonstrate high small-worldness, suggesting optimal network organisation for tasks requiring both attention and rapid decision-making. In contrast, Pedestrian (1.21) shows the lowest small-worldness, indicating a potential trade-off between local and global efficiency for focused attention on slower, predictable movements.

Overall, these metrics reveal task-specific neural network dynamics. High modularity and clustering during Traffic Light and Motor Vehicle suggest compartmentalised processing for predictable, structured cues. High efficiency during Avoiding Object and Crosswalk underscores the brain’s need to integrate information for quick, adaptive responses. These findings emphasise the brain’s ability to dynamically reorganise its connectivity patterns to meet cognitive demands such as attention, decision-making, and action execution during cycling activities.

## 6. Discussion

### 6.1. fNIRS channel selection for identifying traffic events

To identify the most informative fNIRS channels, we established several guiding principles. First, the pairwise between-event comparisons in time-series signals should exhibit a strong distinction between different events, as indicated by the count of significant pairs (Fig 2). This guideline ensures that the channels capture brain activity patterns specific to each type of event, facilitating reliable detection and interpretation. Similar approaches have been employed to differentiate task-specific brain activation patterns in fNIRS studies [17]. We mainly checked the significant signal correspondences between pairs of events in the 4th and 5th seconds. This is because the hemodynamic response to a stimulus typically peaks around 4–6 seconds after onset due to the delay in neurovascular coupling, where increased neuronal activity leads to changes in blood flow and oxygenation. Focusing on the 4-second window allows for consistent comparison across events, as this is when the brain’s response is most likely to stabilise and become detectable. Therefore, analysing differences between traffic events within this time frame enhances the sensitivity and reliability of detecting neural activation changes.

In our data, Channel 4 shows the highest number of significant results between event channels during the 4^th^ and 5^th^ seconds, with a total of 124, followed by Channel 27 with a total of 86 significant pairs, followed by Channels 26 and 3 with a total of 72 significant pairs. This channel corresponds to the left DLPFC, which has been previously identified as a critical contributor to cognitive control of motor behaviour [57], executive function [58,59], intentional decision making [60] and multitasking [61–65]. In our study, these mechanisms were expected to be engaged for several traffic conditions.

Secondly, we focused on channels where between-participant variability is minimal. Reducing variability across participants ensures consistency in how the targeted brain regions respond to similar events, highlighting the universality of the observed patterns. Studies exploring inter-individual consistency in fNIRS signals during cognitive tasks underscore the importance of minimising variability for generalizable findings [66].

Finally, the variability in hemodynamic activity between events should be maximal. This convergence around distinguishable peaks or patterns enables a few select channels to effectively differentiate cognitive load states associated with various events. Such an approach aligns with research that emphasises the importance of optimising signal-to-noise ratios and task-specific contrasts for effective decoding [67]. Together, these principles aim to optimise the utility of the selected channels for future brain-state decoding applications, balancing robustness, specificity, and efficiency.

### 6.2. Graphical features of prefrontal cortex activity during traffic events

Graphical analysis of oxygenation of prefrontal cortex regions revealed intriguing patterns. The connection between the DLPFC and OFC exhibited the highest strength (0.329), indicating robust interaction during the tasks, likely reflecting active engagement in object identification processes. A previous study revealed that the DLPFC and OFC connection might be associated with self-control [68] and their interaction might reflect a form of reinforcement learning [69,70]. Self-control or self-regulation is shown to be a critical indicator in driving context, which explains that drivers adjust their behaviour while driving by utilising their cognitive, sensory and motor capabilities [71]. On the other hand, reinforcement learning is also another important mechanism in the human brain, which can also be active during cycling. In state perception, individuals continuously process the external stimuli such as traffic lights, horn sounds and speed sense. While selecting the action, the individuals might increase or decrease speed, change line or stop. Reward and punishment might be explained as stopping at a red light and avoiding a fine. In this period, the individual also learn which path has more intense traffic.

The connection within the DMPFC was also strong (0.267), suggesting coordinated activity potentially linked to spatial or attentional processing. A connection between the DLPFC and DMPFC showed a strength of 0.263, highlighting their mutual involvement in the task. DLPFC and DMPFC support cognitive control, where DMPFC is known as responsible for cognitive monitoring and DLPFC for adjusting behaviour, and different regions of DMPFC and DLPFC collaboratively play a role to guide adaptive control of behaviour [72]. The OFC regions displayed notable bilateral interaction with a strength of 0.242, possibly supporting task-related cognitive or motor functions. OFC plays a critical role in sensory integration and decision making [73,74], which are frequently used cognitive functions during cycling. Finally, the VMPFC regions demonstrated consistent inter-regional communication with a connection strength of 0.233, which might contribute to the integration of sensory and motor functions during the task.

These findings highlight specific inter-regional connections in the brain that are prominently active during the task of indicating objects, compared to the baseline state. The identified patterns provide insights into the functional connectivity associated with attention, motor coordination, and sensory integration, as captured by fNIRS.

### 6.3. Limitations and future directions

The hemodynamic response baseline duration (4–5 seconds) requirement is not perfectly aligned with our data due to the real-time analysis nature of our application. However, it is important to note that our stimulus onset times corresponded to the moment the object of the event became visible in the camera recordings attached to the bike. This timing might have been slightly different from when the participant became aware of the event. Since the optode coverage was restricted to the prefrontal, parietal, and frontal pole regions, it remains unclear whether other areas, such as the visual or temporal cortices, were also affected by the load-related increase. Moreover, physiological effects on hemodynamic response could not be removed due to technical limitations. Systemic physiology is a critical component which can be either task-induced or non-task-induced and needs to be removed for more accurate analysis [75]. There are several data-driven approaches that can be used to remove systemic artefacts, such as baseline principal component analysis (PCA) [76] and common average reference filter (CAR) [77]. However, CAR might yield overcorrection in long-distance channels [78], and for PCA, the main assumption is that the systemic activity is available for a significant part of the variance in the data and can be removed by using an initial set of principal components [79]. As Klein et al. [78] suggested, methods using short channels provided better results than methods that do not use them [78]. Due to these reasons, we did not take the risk of overcorrecting the data, and systemic physiology might be available in our hemodynamic response.

While it is intuitive to expect a negative correlation between cognitive load and cycling performance, it’s important to highlight that this study did not assess cycling performance or decision-making related to following traffic rules. Changes in cerebral oxygenation over time may also occur without corresponding changes in performance [80]. Informed by the optimal optodes identified as neuromarkers in this study, practical applications for improving the safety of cyclists can be developed in the future, such as neurofeedback systems integrated into cycling equipment. Viewing a pedestrian and avoiding an object are dynamic route events, and crossing over a crosswalk is a static route event; mostly, dynamic route events coincide with a significant change in cognitive load.

Considering the critical role of cognitive load in potentially hazardous situations, revealing cognitive load changes that cyclists undergo and the manifestation of these changes can help devise measures that proactively improve the safety of cyclists. Future research should further examine the links between cognitive load and traffic safety, including how people react to situations causing high cognitive loading. For instance, in such situations (e.g., mixed traffic, shared space), cyclists might be more aware of risk, reduce speed and effectively reduce crash risk.

## 7. Conclusions

In this study, we investigated neural connectivity patterns, highlighting how different road events elicit varying cognitive and neural demands in cyclists. Events like intersections and pedestrian encounters show dense connectivity, particularly in regions related to decision-making, attention, and motor planning. This suggests that these scenarios place a high cognitive load on cyclists, increasing the likelihood of errors or delayed reactions. Roundabouts and traffic light scenarios demonstrate intermediate connectivity, indicating the need for adaptive attention and action selection. Cyclists must balance spatial awareness, rule-following, and interaction with other road users.

Events like avoiding objects and motor vehicle encounters show sparse yet targeted connectivity, reflecting reliance on reflexive and automated responses. These scenarios are time-critical, and any distraction or delayed reaction could lead to increased cognitive load. Crosswalk scenarios show moderate connectivity, indicating relatively lower cognitive demands in controlled environments. Increased connectivity is predominantly observed during cognitively demanding events, highlighting the prefrontal cortex’s dynamic adaptation to environmental changes. Conversely, decreased connectivity is more prominent in simpler situations, reflecting a downregulation of less essential pathways to prioritise automated processes.

Consequently, this study contributes to understanding the patterns of cognitive load in cyclists during various events. Such understanding has the potential to provide valuable insights into traffic safety by revealing the cognitive processes behind cyclists’ behaviour during traffic events. We showed that functional near-infrared spectroscopy (fNIRS) can be an effective tool for research focusing on the understanding of the cognitive load in cyclists during various traffic events.

## Supporting information

S1 Appendix Supplementary results. (DOCX)

S1 File Supplementary findings based on deoxyhaemoglobin levels. (DOCX)
