# Supplementary material for: Cognitive load in cyclists while navigating in traffic: Effects of static and dynamic route events on neural activity of cyclists measured by fNIRS
Source: PLoS One. 2025 Dec 19;20(12):e0339027. doi: 10.1371/journal.pone.0339027 (PMC12716766; doi:10.1371/journal.pone.0339027)
Supplement: S1 Appendix — (DOCX) [file pone.0339027.s001.docx]

**Appendix**

**
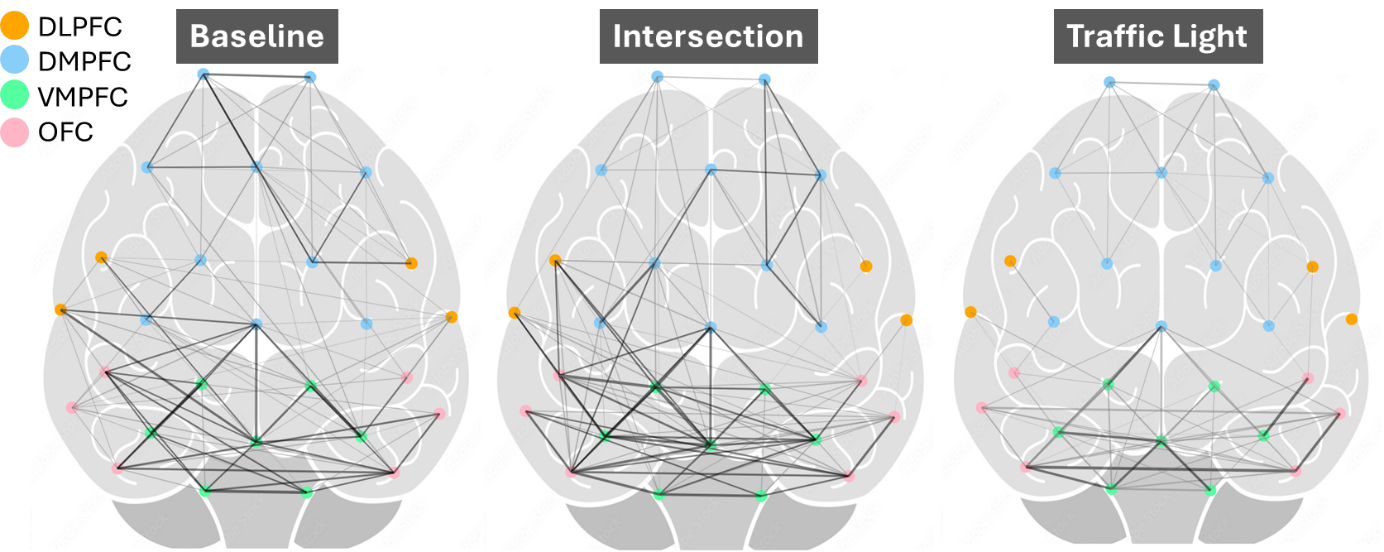
**

**
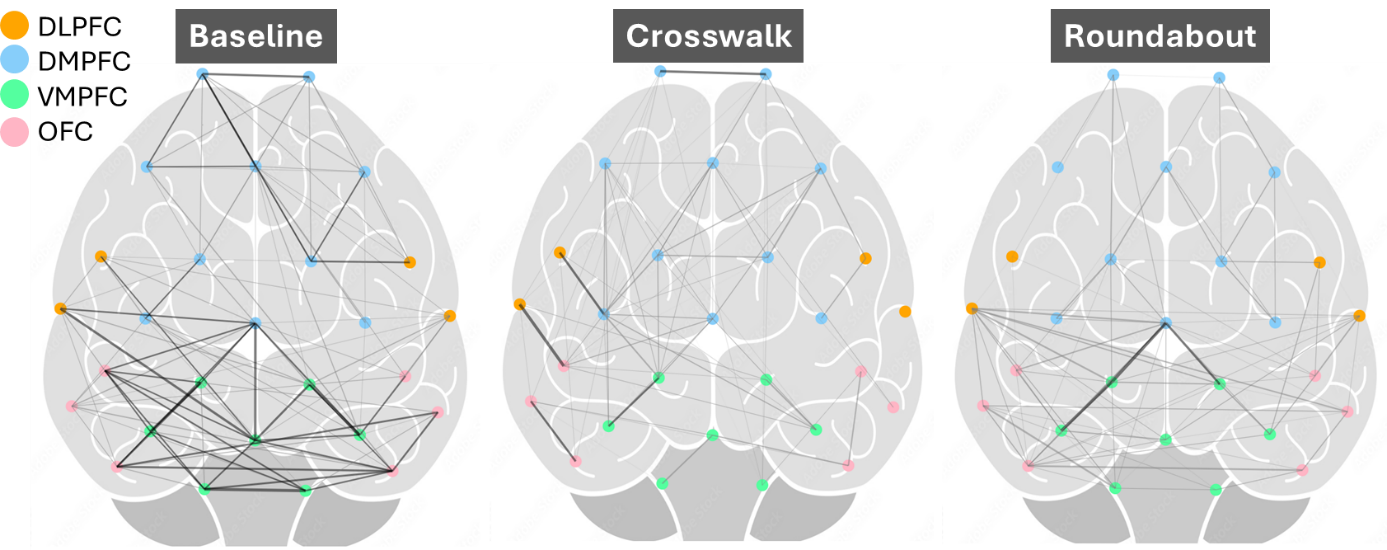
**

**
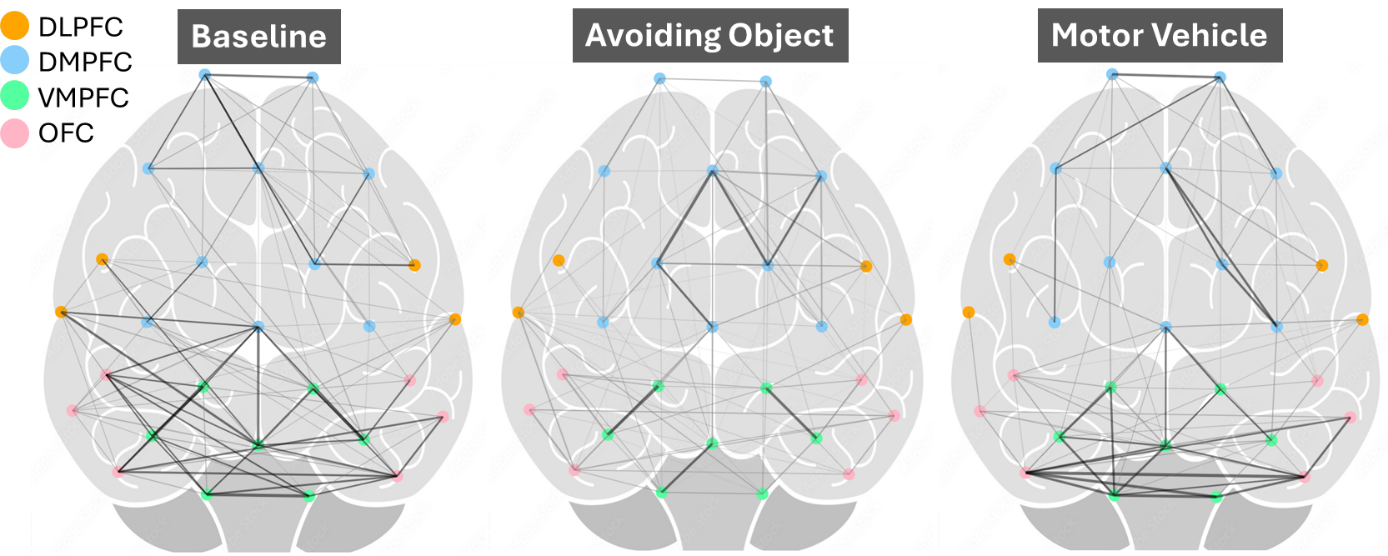
**


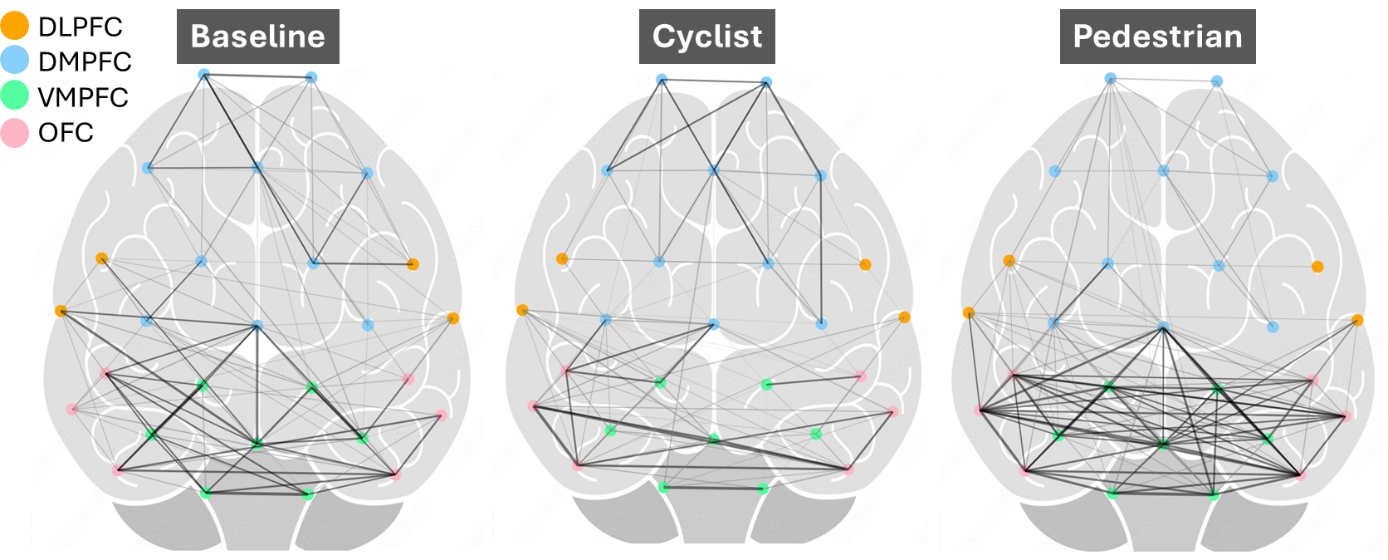


Figure A1. Identified relationship networks for each event and baseline


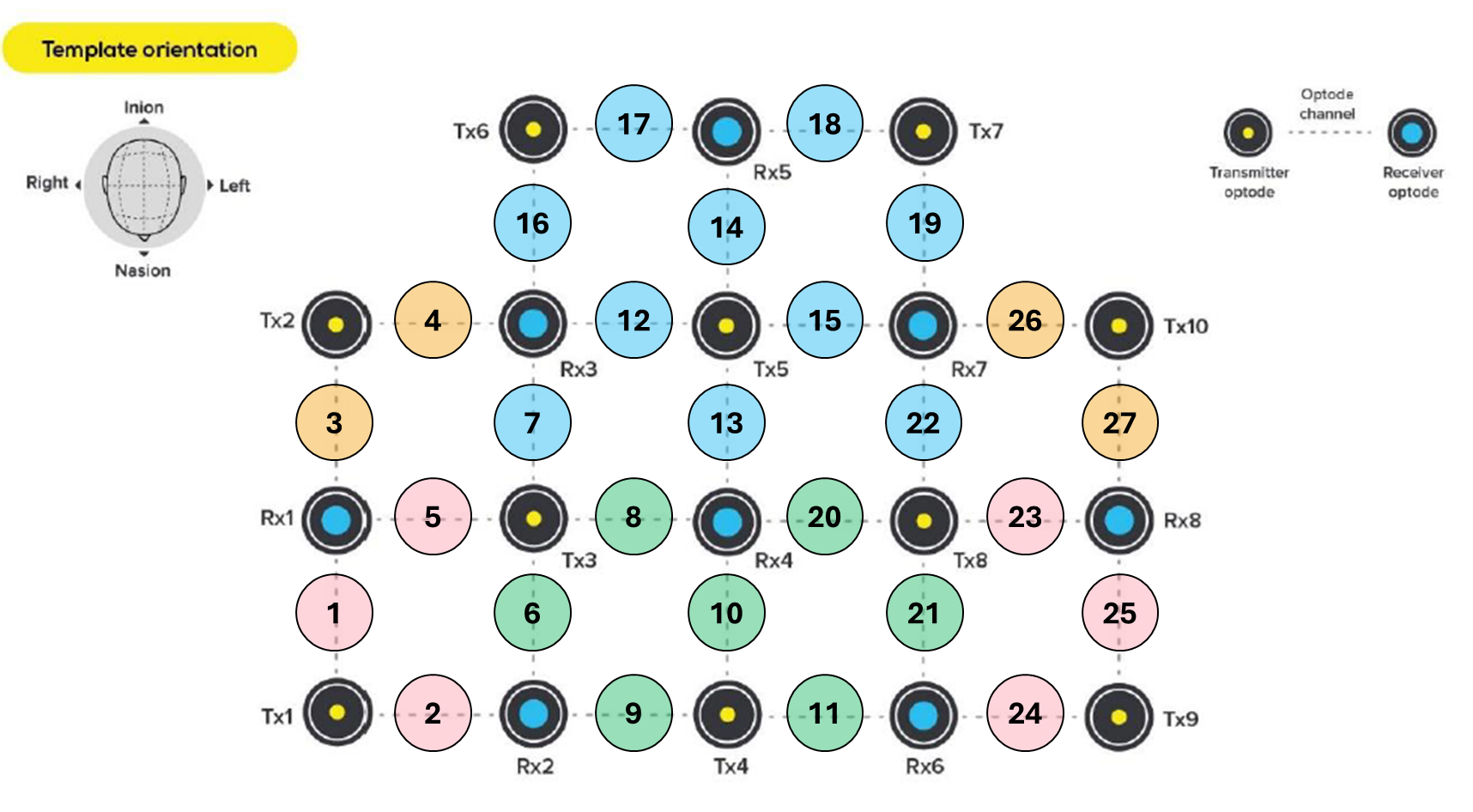


Figure A2. Artinis Optode Template Guide (Artinis Medical Systems, 2024a)

Table A. Channel names, optode position, and corresponding brain region

| **Channel Name** | **Channel position** | **MNI-Coordinate (x y z)** | **Assigned Brain Region** |
| --- | --- | --- | --- |
| Channel 1 | Rx1 - Tx1 (1,1) | -103,07 -59,70 34,75 | OFC |
| Channel 2 | Rx2 - Tx1 (1,2) | -77,65 -82,73 44,43 | OFC |
| Channel 3 | Rx1 - Tx2 (2,1) | -109,32 -22,60 54,85 | DLPFC |
| Channel 4 | Rx3 - Tx2 (2,3) | -86,61 -2,77 84,88 | DLPFC |
| Channel 5 | Rx1 - Tx3 (3,1) | -84,39 -46,20 74,14 | OFC |
| Channel 6 | Rx2 - Tx3 (3,2) | -58,97 -69,23 83,82 | VMPFC |
| Channel 7 | Rx3 - Tx3 (3,3) | -61,69 -26,37 104,18 | DMPFC |
| Channel 8 | Rx4 - Tx3 (3,4) | -30,57 -50,71 106,84 | VMPFC |
| Channel 9 | Rx2 - Tx4 (4,2) | -28,58 -91,20 76,87 | VMPFC |
| Channel 10 | Rx4 - Tx4 (4,4) | -0,17 -72,68 99,89 | VMPFC |
| Channel 11 | Rx6 - Tx4 (4,6) | 28,07 -91,81 76,31 | VMPFC |
| Channel 12 | Rx3 - Tx5 (5,3) | -31,18 -3,80 117,85 | DMPFC |
| Channel 13 | Rx4 - Tx5 (5,4) | -0,05 -28,13 120,51 | DMPFC |
| Channel 14 | Rx5 - Tx5 (5,5) | 0,06 31,50 116,40 | DMPFC |
| Channel 15 | Rx7 - Tx5 (5,7) | 31,18 -4,61 117,82 | DMPFC |
| Channel 16 | Rx3 - Tx6 (6,3) | -60,95 31,32 99,58 | DMPFC |
| Channel 17 | Rx5 - Tx6 (6,5) | -29,71 66,62 98,14 | DMPFC |
| Channel 18 | Rx5 - Tx7 (7,5) | 30,04 65,50 98,81 | DMPFC |
| Channel 19 | Rx7 - Tx7 (7,7) | 61,15 29,39 100,22 | DMPFC |
| Channel 20 | Rx4 - Tx8 (8,4) | 30,30 -51,47 106,55 | VMPFC |
| Channel 21 | Rx6 - Tx8 (8,6) | 58,53 -70,60 82,97 | VMPFC |
| Channel 22 | Rx7 - Tx8 (8,7) | 61,54 -27,94 103,85 | DMPFC |
| Channel 23 | Rx8 - Tx8 (8,8) | 83,96 -48,29 73,26 | OFC |
| Channel 24 | Rx6 - Tx9 (9,6) | 76,80 -84,31 42,76 | OFC |
| Channel 25 | Rx8 - Tx9 (9,8) | 102,23 -62,00 33,05 | OFC |
| Channel 26 | Rx7 - Tx10 (10,7) | 86,60 -5,03 84,79 | DLPFC |
| Channel 27 | Rx8 - Tx10 (10,8) | 109,02 -25,37 54,20 | DLPFC |
