# Supplementary material for: Cognitive load in cyclists while navigating in traffic: Effects of static and dynamic route events on neural activity of cyclists measured by fNIRS
Source: PLoS One. 2025 Dec 19;20(12):e0339027. doi: 10.1371/journal.pone.0339027 (PMC12716766; doi:10.1371/journal.pone.0339027)

**Supplementary findings based on deoxyhaemoglobin levels**

The results of the analysis using deoxyhaemoglobin levels show that pairwise events in ΔHbR mostly differed between the 2- 3 sec and connectivity among regions for different events did not show a dense connectivity pattern unlike ΔHbO data. We think that due to having low signal to noise ratio compared to ΔHbO, ΔHbR did not provide a clear difference across conditions.


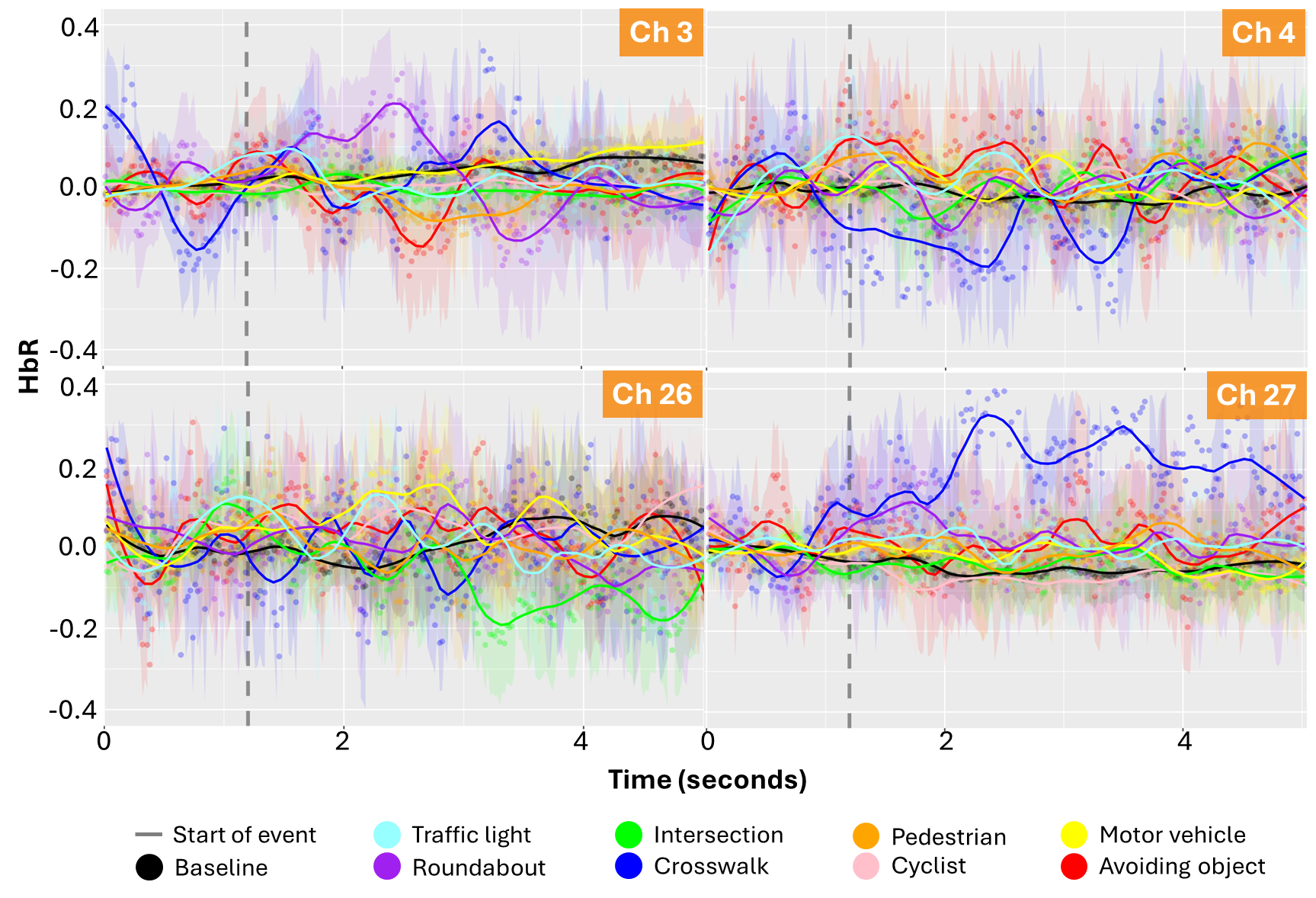


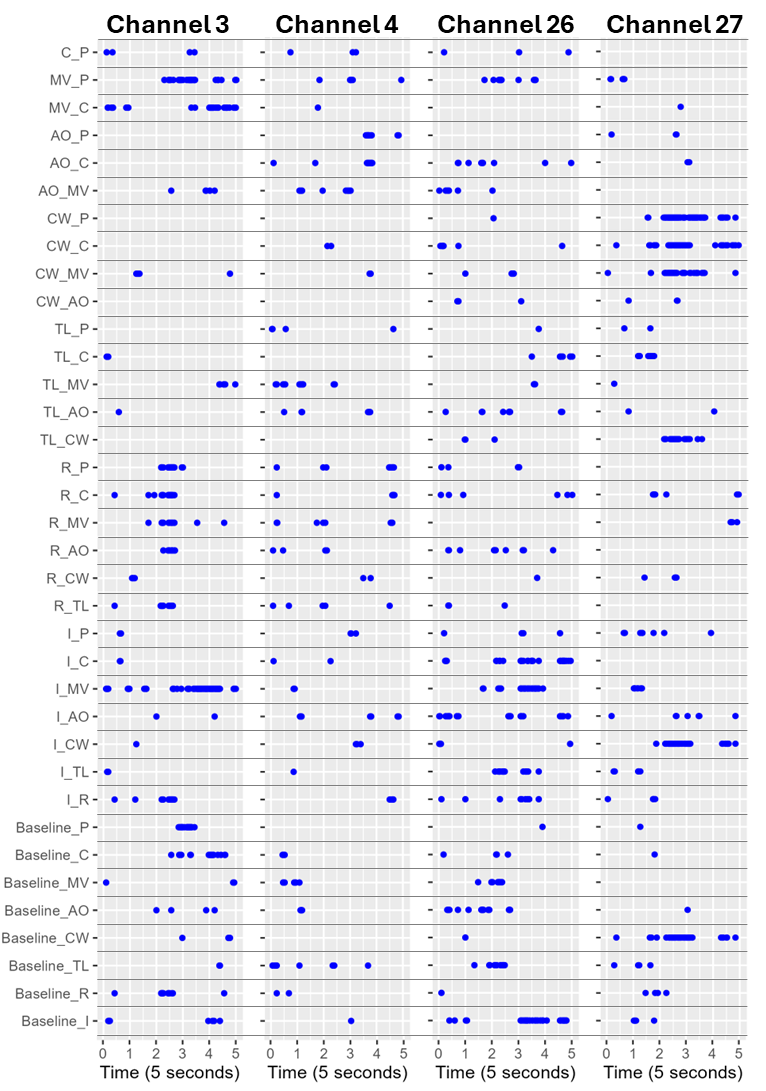


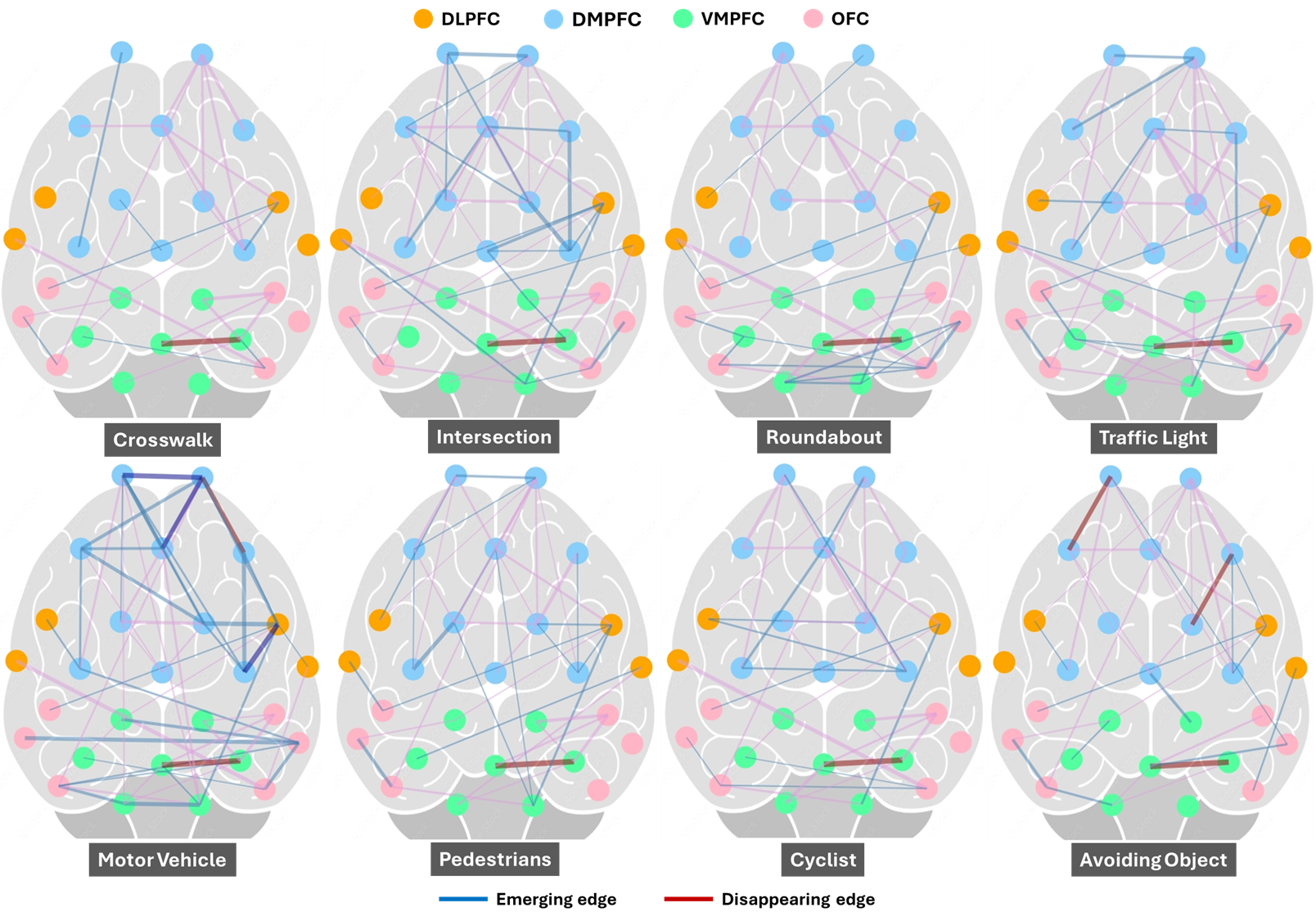

Supplement: S1 File — (DOCX) [file pone.0339027.s002.docx]
